# The Perception of Illness in People with Advanced Chronic Kidney Disease

**DOI:** 10.3390/jpm15030120

**Published:** 2025-03-20

**Authors:** Miquel Sitjar-Suñer, Rosa Suñer-Soler, Afra Masià-Plana, Bernat Carles Serdà-Ferrer, Xavier Pericot-Mozo, Glòria Reig-Garcia

**Affiliations:** 1 Primary Health Centre, Institut Català de la Salut, 17800 Olot, Spain; miquel.sitjar@udg.edu; 2 Nursing Department, University of Girona, 17003 Girona, Spain; afra.masia@udg.edu (A.M.-P.); bernat.serda@udg.edu (B.C.S.-F.); gloria.reig@udg.edu (G.R.-G.); 3 Health and Health Care Research Group, Department of Nursing, University of Girona, 17003 Girona, Spain; 4 Hospital Universitari Dr. Josep Trueta, Institut Català de la Salut, 17007 Girona, Spain

**Keywords:** chronic kidney disease, nurses, illness perception, health status, multicenter study

## Abstract

**Background/objectives**: Chronic kidney disease (CKD) has become an important public health issue; however, there are few investigations regarding the perception of CKD in its advanced stages. Personalized medicine approaches, which take into account knowledge of the disease, symptoms and treatment responses, can improve the perception of the disease and help control the progression of CKD. This study aimed to describe illness perception in people with advanced CKD in primary healthcare settings. **Methods**: A cross-sectional and multicenter descriptive study was conducted amongst a sample of 189 people over 18 years of age with advanced CKD and a glomerular filtration rate between 15 and 29 mL/min/1.73 m^2^ in three community health centers, including rural and urban areas, during 2023. Data on sociodemographic and clinical variables were collected through an ad hoc questionnaire and those on the perception of disease through the Brief Illness Perception Questionnaire. Nurses at the centers collected data from the study. **Results**: The mean age was 79.7, and all participants suffered from another chronic condition in addition to CKD. The mean total score for perception of the disease was 44.02 points, and the dimensions of the duration of treatment control and understanding had the highest evaluations. Men had a greater perception in the dimensions of concern (*p* = 0.023) and understanding (*p* = 0.006). The dimension of consequences showed a correlation with identity (Spearman’s Rho 0.688; *p* = 0.001), and concern about the disease was associated with emotional response (Spearman’s Rho 0.689; *p* < 0.001). A higher number of hospital admissions was associated with a higher score on the questionnaire (B = 4.93; *p* < 0.001; CI: 3.01–6.84) in a multiple linear regression. **Conclusions**: Participants in this study with advanced CKD had low illness perception; women expressed less concern in understanding their health status. Higher symptom burden was linked to greater illness perception, greater emotional impact, and increased hospital admissions.

## 1. Introduction

Chronic kidney disease (CKD) has become an important public health issue since its complications and expense place a heavy burden on health resources [1]. According to the review study by Kovesdy (2022) [2], conducted on population samples from different countries and regions, and the EPIRCE study conducted with the Spanish population [3], the average prevalence of CKD is around 10–15% in the adult population and more than 30% in people over 65 years old [4]. CKD, from the initial stages, causes a significant increase in the risk of morbidity and overall mortality, both in the general population and at-risk groups [5]. In patients treated in primary care with associated chronic conditions, the prevalence of CKD can reach figures of 35–40% [3]. Chronic kidney disease is characterized by the presence of a persistent structural or functional renal alteration that lasts for more than 3 months, with a decrease in glomerular filtration below 60 mL/min/m^2^ [6,7]. However, as nephron destruction progresses and the glomerular filtration rate falls below 30 mL/min/m^2^, various signs and symptoms, which are usually non-specific, such as fatigue, dizziness, nausea, anorexia and concentration deficits, may begin to appear [7,8]. Nevertheless, kidneys have the ability to compensate for the loss of renal function, meaning that clear symptoms may not manifest until the later stages of the disease [9]. This fact may cause the person not to associate the symptoms with kidney problems [10]. The lack of awareness about the symptoms and consequences of the disease in affected individuals can lead to a delay in adopting preventive measures until irreversible renal replacement therapy is required [11].

In recent years, one of the psychological aspects that has gained attention in chronic disease research relates to the concept of illness awareness, a construct developed from the model of feeling and self-regulation; according to this model, each person organizes, explains, and describes information variably, depending on their experiences, situations, and values [12,13]. Thus, illness perception is related to the cognitive representation and set of beliefs regarding the pathology, symptoms, and treatment [14,15]. Age also influences the acceptance of chronic kidney disease. It has been observed that people under the age of 60 tend to deny the disease more, which may hinder the effectiveness of interventions [16].

The association with other diagnosed chronic conditions, such as diabetes or hypertension, negatively influences the perception of kidney disease as a person may experience a general sense of discomfort without being able to clearly identify the underlying pathology [17]. On the other hand, optimal financial support is a variable that positively influences disease adaptation by improving the process of acquiring coping strategies [18].

It is also known that social support plays an important role in the process of adaptation to chronic illness, acting as a cushion against the effects of psychosocial and physical stress on individuals’ health. Additionally, it can prevent the emergence of adverse psychological effects and stress-related emotional responses [19,20].

The various variables discussed above form part of a complex, ambiguous, multidimensional concept that is open to different interpretations, blending aspects related to social networks, educational level, social class, morbidity, disease duration, and others. While several publications have reported poorer quality of life and greater perception of illness in people undergoing renal replacement therapy, to the best of our knowledge, few studies have addressed the perception of chronic kidney disease in the advanced stages before the need for renal replacement therapy [21]. Given this knowledge gap, studies are needed to explore the perception and awareness of kidney disease in people in the pre-dialysis stages, in order to develop specific interventions targeting modifiable individual variables, promoting adaptation to the disease, alleviating symptoms, and preventing the accelerated progression of renal failure. Additionally, these studies could contribute to designing individualized intervention strategies to help people cope with the disease progression and improve future planning of the most suitable renal replacement therapy based on their goals and lifestyles [22,23].

## 2. Materials and Methods

### 2.1. Study Design

A cross-sectional, multicenter descriptive study was conducted between March and July 2023 in 18 community health centers (CHC) in 3 basic health areas (BHAs) encompassing both rural and urban settings, managed together with [REDACTED].

### 2.2. Setting and Participants

The study included all individuals with advanced CKD and assigned basic care units (physician and nurse). Inclusion criteria were adults over 18 years old with advanced CKD and glomerular filtration rates between 15 and 29 mL/min/m^2^ based on the most recent laboratory results documented in the [REDACTED] medical records. Individuals with advanced cognitive impairment or in a terminal situation were excluded.

### 2.3. Data Collection

**Figure 1:**
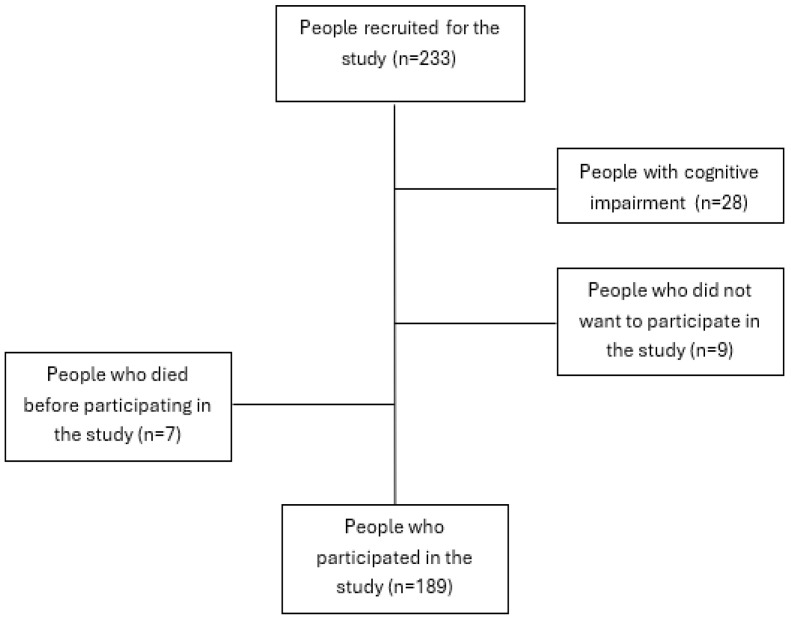
Recruitment process flowchart.

The sample was obtained from a list of people included in one of the three selected primary care areas who met the inclusion criteria (Figure 1). The list corresponds to the total number of outpatients belonging to the three primary care areas of the study with patients who met the inclusion criteria (over 18 years of age, glomerular filtration rate from 15 to 29). Once the sample was selected, nurses contacted each participant by phone to explain the study and request their participation. Those who accepted were contacted by one of the five expert collaborating investigators to arrange an in-person appointment at their healthcare center or home based on their preferences. The survey was self-administered and, on average, took about 20 min to complete. Clinical data were extracted from the patient’s electronic health record. In order to maintain anonymity, a numerical code was assigned in the data collection notebooks, known only to the principal investigator. The confidentiality of participants was ensured during data collection. The confidentiality of participants’ responses was ensured during data collection.

### 2.4. Instruments

Sociodemographic variables (age, sex, employment status, and education level), disease-related variables (morbidity, duration of illness, hospitalizations during the last year) and variables related to illness perception were analyzed using a specific questionnaire.

All variables were collected through an ad hoc questionnaire, and illness perception was assessed using the Brief Illness Perception Questionnaire (BIPQ) [24], validated and translated into Spanish by Pacheco et al. (2012) [25]. This questionnaire comprises 8 items, each rated on a Likert-type scale from 0 (minimum) to 10 (maximum). The initial five items evaluate cognitive perception, including effect on life (item 1); duration of illness (item 2); control over illness (item 3); beliefs about the effectiveness of treatment (item 4); and experience of symptoms (item 5). Items 6 and 8 gauge emotional aspects, specifically concerns related to the illness and a comprehensive assessment of mood. Item 7 evaluates the depth of understanding of the illness. The total score is obtained by adding the results of the 8 items. This score allows for the classification of illness perception into low threat (less than 42 points), moderate threat (between 42 and 49 points) and high threat (more than 50 points). The first five items evaluate cognitive perceptions, while items 6 and 8 evaluate emotional aspects, and item 7 assesses illness understanding [24]. The inter-rater reliability of this instrument was measured with Cohen’s kappa coefficient and found to be 0.92 [26]. In CKD, knowledge of the variables associated with the perception of the disease can be pivotal to the design of personalized intervention strategies. These help people to recognize the disease, which facilitates the optimization of specific intervention to control the progression of CKD and optimize the use of therapeutic resources.

### 2.5. Ethical Considerations

The study was conducted in accordance with the Declaration of Helsinki and approved by the Jordi Gol Ethics Committee (protocol code 22/220-P; date of approval 26 October 2022). The provisions of Organic Law 3/2018 on data protection and digital rights guarantee were adhered to. The ethical principles of the Declaration of Helsinki by the World Medical Association for medical research involving human subjects were followed. Prior to being surveyed, all individuals were informed about the research, received the study information sheet, and provided informed consent. Confidentiality was ensured through anonymization of the data collection notebooks.

### 2.6. Data Analysis

Data were analyzed using the SPSS 27.0 statistical software package (Chicago, IL, USA). Quantitative variables were expressed as mean and standard deviation. Categorical variables were presented as absolute frequency and percentage. The chi-square test and Fisher’s exact test were used to study associations between categorical variables. The comparison of quantitative variables was assessed using non-parametric tests, the Mann–Whitney U test and the Kruskal–Wallis test for independent samples. A multiple linear regression model was used to study variables associated with illness perception. A *p*-value < 0.05 was considered to be statistically significant with a 95% confidence interval.

## 3. Results

### 3.1. Demographic Characteristics

There were 189 participants with a mean age of 79.7 years (DS 12.6), with men being younger than women, with mean ages of 75.9 (SD 12.16) and 82.9 years (SD 12.18), respectively. All participants had another chronic disease in addition to renal disease, with arterial hypertension being the most common (89.9%), followed by heart disease (52.4%) and diabetes (41.3%). Other characteristics of the participants are detailed in Table 1.

### 3.2. Illness Perception Assessment

Regarding the results on illness perception, the specific Brief Illness Perception Questionnaire demonstrated an internal consistency in this study of 0.814 with Cronbach’s alpha. When comparing the items of the questionnaire (Table 2), it was observed that emotional response, identity and consequences were the items with the lowest scores, indicating a less threatening perception of being ill. In contrast, items related to duration, treatment control and understanding of the illness showed higher scores, suggesting a greater perception of illness. Men had higher scores on the items of concern and understanding of illness compared to women (*p* = 0.023 and *p* = 0.006, respectively).

In relation to the results on illness perception assessed using the Brief Illness Perception Questionnaire, a mean total score of 44.02 points was obtained, with lower scores in women, although without significant differences (Table 2).

When relating the items of the Brief Illness Perception Questionnaire to educational level, it was observed that individuals with tertiary education obtained slightly lower scores in the items of identity (*p* = 0.016). Furthermore, those with secondary and/or tertiary education showed higher scores in the item of understanding of illness (*p* = 0.032) (Table 3).

When relating the perception of illness classified into low, moderate and high threat categories, it was not associated with age or clinical variables related to glomerular filtration rate or years of disease progression (Table 4).

When correlating specific items of illness perception, it was seen that the consequences item mainly correlated with identity (Spearman’s Rho 0.688; *p* = 0.001), where higher scores on this item (indicating a greater impact of the disease on daily life) were associated with higher scores on the identity component. With regard to the concern about the illness component, it was found to mainly be correlated with emotional response, with higher scores (indicating more concern about the illness) being associated with higher scores in emotional response (Spearman’s Rho 0.689; *p* < 0.001).

Finally, a multiple linear regression model was constructed to study variables strongly associated with illness perception. Taking the total score of the Brief Illness Perception Questionnaire as the dependent variable, it was observed that only the number of hospital admissions over the previous year was significantly associated with this perception. A higher number of hospital admissions was associated with a higher score on the questionnaire (B = 4.93; *p* < 0.001; CI: 3.01–6.84). With regard to the linear regression, we performed the Durbin–Watson test for detecting the presence of autocorrelation in the residuals (prediction errors) of a regression analysis, which in our analysis was 1.727 (Table 5).

## 4. Discussion

This study analyzed illness perception in people with advanced CKD before initiating dialysis. The sample included 189 participants, mostly women aged 65 years or older. The higher percentage of women in this research is consistent with the recent systematic review by Hill et al. (2016) [27] indicating a higher prevalence of CKD in women, as well as with other national studies [28]. However, other research has not pointed to sex differences [29]. Our results may be attributed to the age disparity between women and men, with women being, on average, nearly 7 years older, resulting in a higher percentage of women due to their greater survival. The mean age of 79.7 years among our participants exceeds that of most studies, which typically describe disease prevalence around 60 years [30]. However, it should be noted that disease prevalence is related to physiological decline in glomerular filtration and associated comorbidity [31]. This disease progression with age could explain the difference in participant age found in our study, as individuals in very advanced stages of CKD prior to dialysis, according to the KDIGO classification (2013) [32], were recruited.

All of our participants had at least one associated chronic disease. Several studies identify hypertension, diabetes and cardiovascular diseases as risk factors for CKD [30,32,33]. Although most participants were retired due to their age, different studies indicate that CKD symptoms can impede work activity even in those of working age [34,35].

Several authors report disease progression times similar to or even longer than those found in this study [36,37]. The majority were not hospitalized during the previous year, similar to the data provided by Anderegg et al. (2018) [38], who described that the care and management of these patients are predominantly conducted on an outpatient basis.

People with more symptoms exhibited a greater illness perception [39]. Additionally, those with higher levels of concern showed greater emotional impact. Many studies find that health status influences illness perception [40,41]. The overall illness perception score from the Brief Illness Perception Questionnaire [24] was similar to that of other studies with patients with chronic diseases where perception was not negative [42,43]. However, according to Hsiao et al. (2023) [44], the perception of threat of illness increases significantly when people require dialysis to survive. This could explain why CKD in stages prior to dialysis is not perceived as threatening but is when dialysis is initiated.

When analyzing the questionnaire items, greater perceived threat was observed in the items related to disease duration, treatment control, and disease understanding. We have not found similar studies in people with advanced CKD to compare these results. The items with lower scores for perceived threat were emotional response, identity and consequences, which is a finding that is similar to those obtained in various studies involving people with other chronic diseases [42,45,46]. There were no sex-based differences in the overall disease perception score; however, in the concern and disease understanding item, males scored higher than females with significant differences, possibly due to men’s limited engagement and experience with the disease, given the historical responsibility of women in caregiving for those in need. Hansen et al. (2021) [47] reported more disease concern in men with higher education levels and greater comorbidity. Regarding disease understanding, no studies differentiating by sex have been found, although distinctions have been observed with other variables such as educational level [48]. In our context, this difference could possibly be explained by the traditional role of women in providing support, attention, and care during illness processes [49].

Participants with secondary and tertiary education demonstrated better disease understanding, lower disease perception, reduced perception of CKD symptoms, and less emotional distress. Additionally, they exhibited greater confidence in the effectiveness of the proposed medical treatment compared to those with primary education. Recent research emphasizes the benefits of educational attainment to the health of individuals with chronic diseases, including improved quality of life and reduced morbidity and mortality [44,49,50,51]. Regarding confidence in the healthcare system, Coats et al. (2018) [52] observed that people with lower education levels tended to display greater trust in healthcare personnel.

The relationship between the perception of bothersome disease symptoms and the impact of disease on daily life observed in our participants aligns with findings from several studies. These studies conclude that as the perception of symptoms characterized by physical discomfort such as pain or fatigue increases, there is a greater impact on the ability to carry out everyday activities, such as work, personal care, social relationships, and leisure activities [53,54]. Therefore, the perception of symptoms resulting from CKD, taken together with our knowledge of how the disease affects daily life, allows us to conclude that the implementation of symptom management strategies and support to minimize the effects of CKD are actions that are of paramount importance. The association between disease concern and the emotional component has also been observed in other studies [55]. This link could be explained by the interconnection of physical, psychological, and social dimensions [56], underscoring the importance of addressing both physical and emotional symptoms in managing this disease. Additionally, a higher number of hospital admissions is directly related to a greater perception of disease, as hospitalizations due to health complications exacerbate symptoms and disease awareness in individuals with CDK [57].

With regard to limitations, although the emotional perception of illness was included, a possible limitation could be the lack of consideration of other psychological factors, such as anxiety or depression, which were not specifically addressed, and which could also influence the overall perception of health and the general well-being of patients.

The overall findings regarding disease perception have implications for improving nursing care for people affected by CKD, indicating the need to place greater emphasis on mental health and empower these people to manage their emotions in order to better cope with and navigate this disease. Furthermore, the implementation of personalized interventions in the follow-up and treatment of CKD could help patients better understand the process of the disease. This approach would enable the identification of possible complications, the adaptation of the treatment to individual needs and, even, the delay in the need to initiate renal replacement therapy.

## 5. Conclusions

The sample consisted mostly of women who have had CKD for more than 5 years. All participants had at least one other chronic illness, with arterial hypertension being the most common, followed by heart disease and diabetes. The illness perception according to the Brief Illness Perception Questionnaire was moderate. The items related to emotional response, identity and consequences obtained lower scores, indicating a lower perception of illness, while the items related to duration, treatment control and understanding of illness obtained high scores, showing a greater perception of illness. Additionally, men had higher scores for the items of concern about the illness and understanding of the illness. The number of hospital admissions in the last few years was associated with a higher illness perception, indicating that those with more hospital admissions scored higher on the illness perception questionnaire. Therefore, it is necessary to place emphasis on health education to help people with CKD identify emotional responses and other effects associated with the disease.

## Figures and Tables

**Table 1:** Characteristics of the participants (n = 189).

**Sex**	
Men	86 (45.5)
Women	103 (54.5)
**Educational level**	
Illiterate	6 (3.2)
Without studies (knows how to read and write)	81 (42.9)
Primary studies	64 (33.9)
Secondary studies	26 (13.7)
Tertiary education	12 (6.3)
**Employment situation**	
Employed	17 (9)
Unemployed	15 (7.9)
Retired	157 (83.1)
**Glomerular filtration**	24 (4.1)
**Years of disease progression**	
5 or less years	35 (18.5)
6 or more years	154 (81.5)
**Number of hospital admissions last year (SD)**	0.67 (1.1)

Note: The quantitative variables are described with the mean and its standard deviation in parentheses, and the qualitative variables are described with the absolute frequency and its percentage by columns.

**Table 2:** Brief Illness Perception Questionnaire scores.

Specific Scale to Determine the Perception of Illness, *Brief* *Illness Perception Questionnaire*	Total Population n: 189 Mean (SD)	Men n 86 Mean (SD)	Women n: 103 Mean (SD)	*p*
Consequences	5.03 (3.04)	5 (3)	5.06 (3.09)	0.929
Timeline	7.28 (3.07)	7.55 (2.81)	7.05 (3.26)	0.435
Personal control	5.24 (3.14)	5.47 (3.07)	5.05 (3.20)	0.376
Treatment control	6.99 (2.55)	7.28 (2.54)	6.76 (2.55)	0.062
Identity	4.60 (3.35)	4.56 (3.48)	4.64 (3.25)	0.988
Concern	5.12 (3.22)	5.69 (3.14)	4.65 (3.22)	0.023
Understanding	5.90 (2.98)	6.52 (2.85)	5.39 (3)	0.006
Emotional response	3.85 (3.29)	3.94 (3.24)	3.77 (3.34)	0.597
Total score	44.02 (16.30)	46 (16.11)	42.36 (16.35)	0.073

**Table 3:** Relationship between education level and perception of illness.

Questionnaire *Brief Illness Perception Questionnaire*	Educational Level			*p*
	Primary	Secondary	Tertiary	
	n: 151	n: 26	n: 12	
	Mean (SD)	Mean (SD)	Mean (SD)	
Consequences	5.26 (3.01)	4.35 (3.38)	3.58 (1.97)	0.199
Timeline	7.17 (3.05)	7.81 (3.17)	7.42 (3.17)	0.409
Personal control	5.03 (3.20)	5.92 (2.92)	6.42 (2.42)	0.250
Treatment control	6.78 (2.62)	8.08 (2.05)	7.33 (2.27)	0.110
Identity	4.77 (3.35)	4.42 (3.46)	2.92 (2.74)	0.016
Concern	5.12 (3.20)	5.65 (3.40)	4 (3.04)	0.214
Understanding	5.62 (2.94)	7.35 (2.71)	6.42 (3.31)	0.032
Emotional response	4 (3.36)	3.58 (3.06)	2.5 (2.71)	0.625
Total score	43.75 (16.69)	47.15 (14.91)	40.58 (14.20)	0.349

**Table 4:** Relationship between illness perception with age, years of evolution, and glomerular filtration rate.

	Low Disease Threat n: 82 Mean (SD)	Moderate Disease Threat n: 26 Mean (SD)	High Disease Threat n: 80 Mean (SD)	*p*
Age	79.29 (13.46)	80.27 (12.11)	80.05 (12.04)	0.886
Years of disease evolution	11.57 (8.86)	11 (6.32)	11.83 (8.31)	0.908
Glomerular filtration rate	23.93 (4.15)	23.39 (4.74)	24.25 (3.91)	0.649

**Table 5:** Multiple linear regression model to study factors associated with illness perception.

Dependent Variable	Independent Variable	B	SE	t	*p*	CI (95%)
Total score of the Brief Illness Perception Questionnaire	Age	−0.13	0.11	−1.13	0.26	(−0.37–0.10)
	Sex	−4.12	2.31	−1.78	0.07	(−8.68–0.43)
	Educational level	−1.12	1.24	−0.90	0.36	(−3.57–1.32)
	Employment situation	3.77	2.48	1.52	0.13	(−1.12–8.67)
	Years of disease progression	0.11	0.15	0.74	0.45	(−0.19–0.42)
	Glomerular filtration	0.02	0.27	0.08	0.93	(−0.51–0.56)
	Number of hospital admissions last year	4.93	0.97	5.08	<0.001	(3.01–6.84)

## Data Availability

The data presented in this study are available upon request from the corresponding author.

## References

[1] Levin A., Stevens P.E., Bilous R.W., Coresh J., De Francisco A.L.M., De Jong P.E., Griffith K.E., Hemmelgarn B.R., Iseki K., Lamb E.J. (2013). Kidney Disease: Improving Global Outcomes (e) CKD Work Group. KDIGO 2012 clinical practice guideline for the evaluation and management of chronic kidney disease. Kidney Int. Suppl..

[2] Kovesdy C.P. (2022). Epidemiology of chronic kidney disease: An update 2022. Kidney Int. Suppl..

[3] Otero González A., Francisco A.D., Gayoso P., García F. (2010). Prevalencia de la insuficiencia renal crónica en España: Resultados del estudio EPIRCE. Nefrología.

[4] Gorostidi M., Sánchez-Martínez M., Ruilope L.M., Graciani A., de la Cruz J.J., Santamaría R., del Pino M.D., Guallar-Castillón P., de Álvaro F., Rodríguez-Artalejo F. (2018). Chronic kidney disease in Spain: Prevalence and impact of accumulation of cardiovascular risk factors. Nefrología (Engl. Ed.).

[5] Gansevoort R.T., Matsushita K., van der Velde M., Astor B.C., Woodward M., Levey A.S., de Jong P.E., Coresh J. (2011). Lower estimated GFR and higher albuminuria are associated with adverse kidney outcomes. A collaborative meta-analysis of general and high-risk population cohorts. Kidney Int..

[6] Aparcana-Granda D.J., Ascencio E.J., Larco R.M.C. (2022). Systematic review of diagnostic and prognostic models of chronic kidney disease in low-income and middle-income countries. BMJ Open.

[7] San Blas J.C.H., Morffi L.R., Figueredo N.A., Díaz A.S., Ferguson Y.M., Viera Y.P. (2022). Marcadores de daño renal y progresión de la insuficiencia renal crónica en el adulto mayor. Mediciego.

[8] Forbes A., Gallagher H. (2020). Chronic kidney disease in adults: Assessment and management. Clin. Med..

[9] Maremonti F., Meyer C., Linkermann A. (2022). Mechanisms and Models of Kidney Tubular Necrosis and Nephron Loss. J. Am. Soc. Nephrol. JASN.

[10] Weldam S.W., Lammers J.W.J., Decates R.L., Schuurmans M.J. (2013). Daily activities and health-related quality of life in patients with chronic obstructive pulmonary disease: Psychological determinants: A cross-sectional study. Health Qual. Life Outcomes.

[11] Lugo-González I.V., Reynoso-Erazo L., Fernández Vega M. (2014). Illness perception, depression, anxiety and asthma control: A first approximation. Neumol. Cirugía Tórax.

[12] Leventhal H., Brissette I., Leventhal E.A. (2012). The common-sense model of self-regulation of health and illness. The Self-Regulation of Health and Illness Behaviour.

[13] Singh A., Rejeb A. (2024). Illness perception: A bibliometric study. Heliyon.

[14] Clarke A.L., Yates T., Smith A.C., Chilcot J. (2016). Patient’s perceptions of chronic kidney disease and their association with psychosocial and clinical outcomes: A narrative review. Clin. Kidney J..

[15] Muscat P., Weinman J., Farrugia E., Callus R., Chilcot J. (2021). Illness perceptions predict distress in patients with chronic kidney disease. BMC Psychol..

[16] Mehrizi F.Z., Bagherian S., Bahramnejad A., Khoshnood Z. (2022). The impact of logo-therapy on disease acceptance and self-awareness of patients undergoing hemodialysis; a pre-test-post-test research. BMC Psychiatry.

[17] Akca N., Saygili M., Ture A.K. (2022). The relationship between the perception of chronic disease care and health-related quality of life in adults with chronic kidney disease. Chronic Illn..

[18] Van Hecke A., Heinen M., Fernández-Ortega P., Graue M., Hendriks J.M.L., Høy B., Köpke S., Lithner M., Van Gaal B.G.I. (2017). Systematic literature review on effectiveness of self-management support interventions in patients with chronic conditions and low socio-economic status. J. Adv. Nurs..

[19] Lu Y., Zhai S., Liu Q., Dai C., Liu S., Shang Y., Chen C. (2024). Correlates of symptom burden in renal dialysis patients: A systematic review and meta-analysis. Ren. Fail..

[20] Nagel T., Sweet M., Dingwall K.M., Puszka S., Hughes J.T., Kavanagh D.J., Cass A., Howard K., Majoni S.W. (2020). Adapting wellbeing research tools for Aboriginal and Torres Strait Islander people with chronic kidney disease. BMC Nephrol..

[21] Levey A.S., Titan S.M., Powe N.R., Coresh J., Inker L.A. (2020). Kidney disease, race, and GFR estimation. Clin. J. Am. Soc. Nephrol..

[22] Yagi N., Shukunobe T., Nishimura S., Mima A. (2023). Experience and Daily Burden of Patients with Chronic Kidney Disease Not Receiving Maintenance Dialysis or Renal Transplantation. Adv. Ther..

[23] Campbell-Crofts S.J., Roden J. (2018). Primary health care decision making in pre-dialysis chronic kidney disease. Chronic Illn..

[24] Broadbent E., Petrie K.J., Main J., Weinman J. (2006). The brief illness perception questionnaire. J. Psychosom. Res..

[25] Pacheco-Huergo V., Viladrich C., Pujol-Ribera E., Cabezas-Peña C., Núñez M., Roura-Olmeda P., Amado-Guirado E., Núñez E., del Val J.L. (2012). Percepción en enfermedades crónicas: Validación lingüística del Illness Perception Questionnaire Revised y del Brief Illness Perception Questionnaire para la población española. Atención Primaria.

[26] Lukoševičiūtė J., Šmigelskas K. (2020). Causal item of Brief Illness Perception Questionnaire (BIPQ) scale: The main categories. Health Psychol. Res..

[27] Hill N.R., Fatoba S.T., Oke J.L., Hirst J.A., O’Callaghan C.A., Lasserson D.S., Hobbs F.R. (2016). Global prevalence of chronic kidney disease–a systematic review and meta-analysis. PLoS ONE.

[28] Llisterri J.L., Micó-Pérez R.M., Velilla-Zancada S., Rodríguez-Roca G.C., Prieto-Díaz M.Á., Martín-Sánchez V., Barquilla A., Polo-García J., Segura-Fragoso A., Cinza-Sanjurjo S. (2021). Prevalence of chronic kidney disease and associated factors in the Spanish population attended in primary care: Results of the IBERICAN study. Med. Clínica (Engl. Ed.).

[29] Ruiz-Garcia A., Arranz-Martínez E., Iturmendi-Martínez N., Fernández-Vicente T., Rivera-Teijido M., García-Álvarez J.C. (2023). Prevalence rates of chronic kidney disease and its association with cardiometabolic factors and cardiovascular diseases. SIMETAP-CKD study. Clínica Investig. Arterioscler. (Engl. Ed.).

[30] Chen T.K., Knicely D.H., Grams M.E. (2019). Chronic kidney disease diagnosis and management: A review. JAMA.

[31] Lindeman R.D., Tobin J., Shock N.W. (1985). Longitudinal studies on the rate of decline in renal function with age. J. Am. Geriatr. Soc..

[32] Eckardt K.-U., Kasiske B.L. (2013). KDIGO clinical practice guideline for the diagnosis, evaluation, prevention, and treatment of Chronic Kidney Disease-Mineral and Bone Disorder (CKD-MBD). Kidney Int. Suppl..

[33] Agarwal R., Filippatos G., Pitt B., Anker S.D., Rossing P., Joseph A., Kolkhof P., Nowack C., Gebel M., Ruilop L.M. (2022). Cardiovascular and kidney outcomes with finerenone in patients with type 2 diabetes and chronic kidney disease: The FIDELITY pooled analysis. Eur. Heart J..

[34] Kirkeskov L., Carlsen R.K., Lund T., Buus N.H. (2021). Employment of patients with kidney failure treated with dialysis or kidney transplantation—A systematic review and meta-analysis. BMC Nephrol..

[35] Sitjar-Suñer M., Suñer-Soler R., Masià-Plana A., Chirveches-Pérez E., Bertran-Noguer C., Fuentes-Pumarola C. (2020). Quality of life and social support of people on peritoneal dialysis: Mixed methods research. Int. J. Environ. Res. Public Health.

[36] Gonçalves D.L.N., Moreira T.R., da Silva L.S. (2022). A systematic review and meta-analysis of the association between uric acid levels and chronic kidney disease. Sci. Rep..

[37] Heras M., Guerrero M.T., Fernández-Reyes M.J., Muñoz A. (2017). Enfermedad renal «oculta» en ancianos:¿ deja de ocultarse a los 10 años de seguimiento?. Nefrología.

[38] Anderegg M.D., Gums T.H., Uribe L., MacLaughlin E.J., Hoehns J., Bazaldua O.V., Ives T.J., Hahn D.L., Coffey C.S., Carter B.L. (2018). Pharmacist intervention for blood pressure control in patients with diabetes and/or chronic kidney disease. Pharmacother. J. Hum. Pharmacol. Drug Ther..

[39] Kushwaha R., Vardhan P.S., Kushwaha P.P. (2023). Chronic Kidney Disease Interplay with Comorbidities and Carbohydrate Metabolism: A Review. Life.

[40] Knowles S.R., Cook S.I., Tribbick D. (2013). Relationship between health status, illness perceptions, coping strategies and psychological morbidity: A preliminary study with IBD stoma patients. J. Crohn’s Colitis.

[41] Meuleman Y., van der Bent Y., Gentenaar L., Caskey F.J., Bart H.A., Konijn W.S., Bos Q.J.W., Hemmelder M.H., Dekker F.W. (2024). Exploring Patients’ Perceptions About Chronic Kidney Disease and Their Treatment: A Qualitative Study. Int. J. Behav. Med..

[42] Pesut D., Raskovic S., Tomic-Spiric V., Bulajic M., Bogic M., Bursuc B., Peric-Popadic A. (2014). Gender differences revealed by the Brief Illness Perception Questionnaire in allergic rhinitis. Clin. Respir. J..

[43] Schultz W.M., Kelli H.M., Lisko J.C., Varghese T., Shen J., Sandesara P., Quyyumi A.A., Taylor H.A., Gulati M., Harold J.G. (2018). Socioeconomic status and cardiovascular outcomes: Challenges and interventions. Circulation.

[44] Hsiao S.-M., Kuo M.-C., Hsiao P.-N., Moi S.-H., Chiu Y.-W., Wang S.-L., Chen T.-H., Kung L.-F., Hwang S.-J., Lee C.-L. (2023). Shared decision-making for renal replacement treatment and illness perception in patients with advanced chronic kidney disease. BMC Med. Inform. Decis. Mak..

[45] Adrián-Arrieta L., de Tejerina J.C.F. (2018). Autopercepción de enfermedad en pacientes con enfermedades crónicas. Med. Fam. SEMERGEN.

[46] Fanakidou I., Zyga S., Alikari V., Tsironi M., Stathoulis J., Theofilou P. (2018). Mental health, loneliness, and illness perception outcomes in quality of life among young breast cancer patients after mastectomy: The role of breast reconstruction. Qual. Life Res..

[47] Hansen M.H.M., Primdahl J., Riber L.D., Ekholm O.M., Kristensen K.L., Thrysoee L., Thorup C.B., Mols R., Christensen A.V.M., Rasmussen T.B. (2021). Illness perception after heart valve surgery: Differences among men and women. J. Cardiovasc. Nurs..

[48] Martins Girotto P.C., de Lima Santos A., Silva Marcon S. (2018). Conocimiento y actitud frente a la enfermedad de personas con diabetes mellitus atendidas en Atención Primaria. Enfermería Glob..

[49] Bustillo M., Gómez-Gutiérrez M., Guillén A.I. (2018). Los cuidadores informales de personas mayores dependientes: Una revisión de las intervenciones psicológicas de los últimos diez años. Clínica Salud.

[50] Lipsky M.S., Su S., Crespo C.J., Hung M. (2021). Men and oral health: A review of sex and gender differences. Am. J. Men’s Health.

[51] Sarker M.H.R., Moriyama M., Rashid H.U., Rahman M., Chisti M.J., Das S.K., Saha S.K., El Arifeen S., Ahmed T., Faruque A.S.G. (2022). Chronic kidney disease awareness campaign and mobile health education to improve knowledge, quality of life, and motivation for a healthy lifestyle among patients with chronic kidney disease in Bangladesh: Randomized controlled trial. J. Med. Internet Res..

[52] Coats H., Downey L., Sharma R.K., Curtis J.R., Engelberg R.A. (2018). Quality of communication and trust in patients with serious illness: An exploratory study of the relationships of race/ethnicity, socioeconomic status, and religiosity. J. Pain Symptom Manag..

[53] Fletcher B.R., Damery S., Aiyegbusi O.L., Anderson N., Calvert M., Cockwell P., Ferguson J., Horton M., Paap M.C.S., Sidey-Gibbons C. (2022). Symptom burden and health-related quality of life in chronic kidney disease: A global systematic review and meta-analysis. PLoS Med..

[54] Sbidian E., Chaimani A., Guelimi R., Garcia-Doval I., Hua C., Hughes C., Naldi L., Kinberger M., Afach S., Le Cleach L. (2023). Systemic pharmacological treatments for chronic plaque psoriasis: A network meta-analysis. Cochrane Database Syst. Rev..

[55] Walklin C.G., Young H.M., Asghari E., Bhandari S., E Billany R., Bishop N., Bramham K., Briggs J., Burton J.O., Campbell J. (2023). The effect of a novel, digital physical activity and emotional well-being intervention on health-related quality of life in people with chronic kidney disease: Trial design and baseline data from a multicentre prospective, wait-list randomised controlled trial (Kidney BEAM). BMC Nephrol..

[56] Bautmans I., Knoop V., Thiyagarajan J.A., Maier A.B., Beard J.R., Freiberger E., Belsky D., Aubertin-Leheudre M., Mikton C., Cesari M. (2022). WHO working definition of vitality capacity for healthy longevity monitoring. Lancet Healthy Longev..

[57] Bamforth R.J., Chhibba R., Ferguson T.W., Sabourin J., Pieroni D., Askin N., Tangri N., Komenda P., Rigatto C. (2021). Strategies to prevent hospital readmission and death in patients with chronic heart failure, chronic obstructive pulmonary disease, and chronic kidney disease: A systematic review and meta-analysis. PLoS ONE.

